# Lung Ultrasound for the Diagnosis and Management of Neonatal Respiratory Distress Syndrome: A Minireview

**DOI:** 10.3389/fped.2022.864911

**Published:** 2022-04-14

**Authors:** Bin-Bin Guo, Lin Pang, Bo Yang, Cong Zhang, Xiao-Ya Chen, Jia-Bao OuYang, Chang-Jun Wu

**Affiliations:** ^1^Department of Ultrasound, First Affiliated Hospital of Harbin Medical University, Harbin, China; ^2^Department of Ultrasound, Harbin Finance University, Harbin, China; ^3^Department of Ultrasound, Taian Maternal and Child Health Hospital of Shandong Province, Taian, China

**Keywords:** lung ultrasound, respiratory distress syndrome, neonates, diagnosis, management

## Abstract

Lung ultrasound (LUS) is useful for diagnosis of respiratory distress syndrome in neonates. Recently, it has been proved to play an important role in the management of neonatal respiratory distress syndrome (RDS). It is feasible to grade RDS and select therapeutic modalities accordingly by LUS. The treatment also should be adjusted with the change in ultrasound images. In conclusion, LUS is valuable for the diagnosis and management of neonatal respiratory distress syndrome.

## Introduction

Respiratory distress syndrome (RDS) is one of the most common causes of morbidity and mortality in premature infants. Early diagnosis and treatment are important for neonatal respiratory distress syndrome. The diagnosis of RDS used to rely mainly on x-rays. Recently, lung ultrasound (LUS) has been proven to be useful for respiratory management in neonates (1). LUS cannot only diagnose pulmonary diseases but also plays an important role in differential diagnosis, severity classification, and treatment of RDS in neonates. The purpose of this paper is to review the application of lung ultrasound in neonatal respiratory distress syndrome in recent years.

## Data Sources and Searches

In this systematic review and meta-analysis, two researchers (B.B.G. and C.Z.) independently searched PubMed, the Cochrane Library databases, and Embase for articles from inception to 2022. There was no restriction on language, publication date, or country. The reference lists of the included studies were also manually searched.

## Limitations of Traditional Diagnosis of Neonatal Respiratory Distress Syndrome

Imaging is often used to confirm the diagnosis of neonatal respiratory distress syndrome. Chest CT is not convenient for bedside examination, as it has a high cost, and ionizing radiation should not be widely used in neonates. Bedside chest radiography also has ionizing radiation and has a disadvantage of high misdiagnosis rate (2–4).

## The Role of Lung Ultrasound in the Diagnosis of Neonatal Respiratory Distress Syndrome

Lung ultrasound (LUS) is a simple, non-invasive, inexpensive, and reproducible examination. Current evidence supports LUS as a useful imaging alternative for the diagnosis of RDS, and it has a significant diagnostic accuracy for RDS (5–8). Compared with traditional CXR, LUS diagnoses some lung diseases also with higher accuracy and higher specificity (7). A review, indeed, suggests that lung ultrasound can replace chest x-ray in neonates (9). Actually, in some NICUs, CXR has been entirely replaced by LUS for routine use in neonatal wards (10).

## Ultrasonic Manifestations of Neonatal Respiratory Distress Syndrome

According to the Protocol and Guidelines for Point-of-Care Lung Ultrasound in Diagnosing Neonatal Pulmonary Diseases Based on International Expert Consensus, LUS characteristics and diagnostic criteria for respiratory distress syndrome (RDS) of the newborn: (i) Lung consolidations accompanied by air bronchograms are the most important LUS manifestations of RDS, which are characterized by the following: (a) Consolidations are most often observed in the posterior parts of the lungs. The degree of consolidation is related to the severity of the disease. (b) Consolidations are limited only to the region beneath the pleura in mild RDS patients. Conversely, the areas of consolidation may extend to deeper parts of the lung fields in more severe RDS. (c) Usually, consolidations are visible in different lung fields bilaterally. Nevertheless, they may be limited to certain intercostal spaces on one side of the lung. Consolidated areas show an uneven hypoechoic quality, and the boundary with surrounding lung tissue is clear and easy to distinguish. (d) Air bronchograms show dense, speckled, or snowflake-like shapes. (ii) The pleural line is abnormal, and the A-lines disappear. (iii) The non-consolidated zones may appear as AIS. (iv) Of patients, 15–20% may have different degrees of unilateral or bilateral pleural effusion (11, 12).

Consolidation is the most important ultrasound imaging feature of RDS. Subpleural lung consolidation is an important sign to identify the NRDS and transient tachypnea of the newborn (TTN). It is difficult to identify RDS and TTN by early clinical manifestations. The main characteristic of TTN is lung edema without lung consolidations, and it is diagnosed based on the following findings. (i) Mild TTN mainly manifests as AIS and a double lung point. Severe TTN in the acute period mainly manifests as a compact B-line, white lung, or severe AIS, while a double lung point may appear with disease recovery. (ii) Mild or severe TTN is characterized by pleural line abnormalities, A-line disappearance, and different degrees of pleural effusion in one or the bilateral side of the chest. (iii) No consolidation is observed in the lung fields (11, 12). Thus, TTN diagnosis can be ruled out if lung ultrasound observes subpleural lung consolidation signs (13).

Recently, new ultrasonic concepts, such as “ground glass sign” and “snowflake sign,” have been proposed. Ground glass sign is a kind of mild pulmonary consolidation with similar ground glass appearance on ultrasound image but with no obvious air bronchial sign. It is characterized by strong echo near field and gradually weakened echo from near to far field. The parallel scanning of the probe along the intercostal space shows this slight lesion easier. Snowflake sign is a lung consolidation accompanied by obvious air bronchogram sign that resembles a snow pattern on ultrasound imaging. It is characterized by punctate patchy or line air bronchogram sign on ultrasound. Snowflake lung consolidation is considered to be the most specific ultrasound imaging feature of RDS. The presentation of these signs cannot only improve the diagnostic rate of RDS but also provide a basis for the grading of RDS (14).

As we all know, ultrasound is highly sensitive to lung consolidation, but lung consolidation occurs in many lung diseases, such as pneumonia, pulmonary hemorrhage, etc. The LUS characteristics and diagnostic criteria above further clarify the characteristics and ultrasound manifestations of RDS lung consolidation.

## The Role of Lung Ultrasound in the Grading of Neonatal Respiratory Distress Syndrome

Does an infant with respiratory distress syndrome (RDS) need nasal continuous positive airway pressure (NCPAP) support, mechanical ventilation, or surfactant replacement therapy? The choice of therapy depends on the severity of the condition. Early identification and treatment of RDS reduce the risk of death and bronchopulmonary dysplasia (BPD). Studies have shown that infants treated with surfactant therapy within 2–3 h of life are at lower risk of death and BPD compared with infants treated later (15). Therefore, grading RDS early and accordingly administering it is beneficial. Chest x-ray has been proven to correspond to the severity of NRDS (16). Bober et al. state that the stage of ultrasound examination is closely related to the radiation standards of each stage (17). How does ultrasound grade the severity of NRDS?

Liu et al. point out that RDS could be classified into mild, moderate, and severe degrees according to the degree and scope of lung consolidation and whether it caused serious complications. Lung consolidation with mild RDS presented ground glass sign on ultrasound imaging, which also could be observed in the early or recovery period with moderate to severe RDS. Lung consolidation with moderate RDS showed snowflake signs on ultrasound but did not affect all lung zones. Severe RDS has snowflake sign consolidation that affects all lung zones or any degree or extent of consolidation that causes serious complications such as pulmonary hemorrhage, pneumothorax, massive atelectasis, or persistent pulmonary hypertension (18).

Another study also shows that the LUS score and consolidation areas can discriminate RDS from non-RDS and the different grades of NRDS, and predict the application of mechanical ventilation (19). The LUS score correlates inversely with lung aeration. Nonetheless, it should be noted that, although ultrasound specialists believe that the LUS score may be valuable (20), from a clinician's perspective, the score is of little significance. The accuracy and scientificity of the score system have been questioned by some authors. It is considered that LUS score not only cannot be used to accurately assess the severity of neonatal lung disease but also may result in substantial discrepancies (21). Indeed, the results of lung ultrasound score are subjective, inconsistent, and poorly repeatable.

## The Role of Lung Ultrasound in the Treatment of Neonatal Respiratory Distress Syndrome

### The Role of Lung Ultrasound in the Selection of Therapeutic Modalities

The treatment plan is related to the severity of RDS, which could be determined by lung ultrasound combined with clinical manifestations. The treatment also should be adjusted accordingly with the changes in ultrasound images and neonatal conditions.

As we all know, nasal continuous positive airway pressure (NCPAP) is the most common non-invasive respiratory support used for RDS. If the diagnosis is Grade I RDS on LUS, the non-invasive MV can be administered (22, 23). LUS can help in reducing the usage rate of expensive medicaments such as exogenous pulmonary surfactants (24). Infants with a significant respiratory disease often fail NCPAP support and eventually require mechanical ventilation (MV) or surfactant replacement therapy. If the diagnosis is moderate–severe RDS on LUS, exogenous PS should be provided (22, 23).

Application of surfactants at arbitrary thresholds may delay treatment. Therefore, it may be beneficial to identify the need for surfactant treatment early and to give treatment accordingly. LUS can be used accurately to identify the need for surfactant replacement treatment or mechanical ventilation in infants with respiratory distress treated with nasal continuous positive airway pressure (NCPAP) support. The treatment should be adjusted with the change in ultrasound images.

### The Role of Lung Ultrasound in Monitoring the Therapeutic Effect of Neonatal Respiratory Distress Syndrome

It is necessary to monitor the lung changes with RDS, especially in neonates treated with invasive MV. Repeated CXR for infants in a short time is unethical and harmful due to the increased exposure to ionizing radiation. LUS can semiquantitatively assess lung water content, which makes it possible to provide a more accurate detection and localization of the changes in the lungs compared with CXRs.

LUS has proved to be very reliable in monitoring the clinical changes. Gargani et al. show that sonographic abnormalities in the lungs precede PaO_2_/FiO_2_ changes in an animal model. Therefore, LUS can be easily used for the early detection of neonates with RDS before clinical deterioration and improve the clinical progression and prognosis (25, 26). Furthermore, LUS monitoring can significantly shorten the time spent on mechanical ventilation in neonates with RDS (24).

Changes in pulmonary status can be efficiently followed-up by LUS. In a study, 40 neonates with RDS were examined by ultrasound before and after treatment with surfactants. Lung ultrasound results showed that consolidation and B-line decreased in most of the infants with prolonged treatment with surfactants. This study shows that ultrasound can detect changes in the lungs after surfactant treatment in preterm infants and can be used to screen for further treatment (27). Improvements in LUS findings can be often first observed in anterior lung areas. Transition from consolidation to aggregation-induced emission (AIE), AIE to interstitial edema (IE), and IE to a normal LUS pattern or *vice versa* can be seen. On one hand, this LUS quality allows for estimation of the surfactant replacement therapy effect (11). On the other hand, under LUS monitoring, the need for repeated application of PS or not, the time interval of repeated application, and the dose of repeated use can be correctly determined when using PS (24, 25, 28–30).

In a word, LUS plays an important role in monitoring neonatal respiratory distress syndrome therapeutic effect (31).

### The Role of Lung Ultrasound in Monitoring Complications of Neonatal Respiratory Distress Syndrome

RDS can be associated with many complications, including hemorrhage, pneumothorax, pneumonia, atelectasis and bronchopulmonary dysplasia, etc. LUS also enables the detection of lung complications in preterm infants with RDS. In Lovrenski J's study, the diagnostics of pulmonary complications of RDS shows a positive predictive value of 100%, indicating a high reliability of the method in premature infants with RDS (32).

In addition, weaning under ultrasound guidance can significantly reduce ventilatory time. Lung ultrasound is also helpful to resolve the difficulty of mechanical ventilation withdrawal, which often confuses clinicians (33–35). Lung ultrasound is a rapid, non-invasive, repetitive, and reliable tool for predicting the weaning success of ventilated neonates (36).

## Conclusion and Future Directions

As mentioned above, recent research results show that lung ultrasound cannot only make accurate diagnosis of RDS in neonates but also can classify the severity of RDS, monitor and manage treatment, and thereby improve the prognosis of RDS.

Lung ultrasound plays an important role in the diagnosis and management of RDS, but non-standardization of scanning methods, and incorrect instrument operation and parameter adjustment will seriously affect the accuracy of the examination; thus, it is quite important to accept training in LUS to improve interobserver consistency in diagnostic accuracy, reliability, and patient outcomes (37).

## Author Contributions

B-BG, LP, BY, CZ, X-YC, J-BO, and C-JW conceptualized and designed the study, drafted the initial manuscript, and reviewed and revised the manuscript. All authors contributed to the manuscript revision, read, and approved the submitted version.

## Conflict of Interest

The authors declare that the research was conducted in the absence of any commercial or financial relationships that could be construed as a potential conflict of interest.

## Publisher's Note

All claims expressed in this article are solely those of the authors and do not necessarily represent those of their affiliated organizations, or those of the publisher, the editors and the reviewers. Any product that may be evaluated in this article, or claim that may be made by its manufacturer, is not guaranteed or endorsed by the publisher.
